# Renal denervation in an animal model of diabetes and hypertension: Impact on the autonomic nervous system and nephropathy

**DOI:** 10.1186/1475-2840-10-33

**Published:** 2011-04-17

**Authors:** Lucinara D Dias, Karina R Casali, Natalia M Leguisamo, Felipe Azambuja, Martina S Souza, Maristela Okamoto, Ubiratan F Machado, Maria Cláudia Irigoyen, Beatriz D Schaan

**Affiliations:** 1 Instituto de Cardiologia do Rio Grande do Sul/Fundação Universitária de Cardiologia (IC/FUC), Porto Alegre, Brazil; 2 Universidade Federal do Rio Grande do Sul, Endocrine Division HCPA, Porto Alegre, Brazil; 3 Institute of Biomedical Scientes, University of São Paulo, São Paulo, Brazil; 4 Instituto do Coração (INCOR), São Paulo, Brazil

## Abstract

**Background:**

The effects of renal denervation on cardiovascular reflexes and markers of nephropathy in diabetic-hypertensive rats have not yet been explored.

**Methods:**

Aim: To evaluate the effects of renal denervation on nephropathy development mechanisms (blood pressure, cardiovascular autonomic changes, renal GLUT2) in diabetic-hypertensive rats. Forty-one male spontaneously hypertensive rats (SHR) ~250 g were injected with STZ or not; 30 days later, surgical renal denervation (RD) or sham procedure was performed; 15 days later, glycemia and albuminuria (ELISA) were evaluated. Catheters were implanted into the femoral artery to evaluate arterial pressure (AP) and heart rate variability (spectral analysis) one day later in conscious animals. Animals were killed, kidneys removed, and cortical renal GLUT2 quantified (Western blotting).

**Results:**

Higher glycemia (p < 0.05) and lower mean AP were observed in diabetics *vs. *nondiabetics (p < 0.05). Heart rate was higher in renal-denervated hypertensive and lower in diabetic-hypertensive rats (384.8 ± 37, 431.3 ± 36, 316.2 ± 5, 363.8 ± 12 bpm in SHR, RD-SHR, STZ-SHR and RD-STZ-SHR, respectively). Heart rate variability was higher in renal-denervated diabetic-hypertensive rats (55.75 ± 25.21, 73.40 ± 53.30, 148.4 ± 93 in RD-SHR, STZ-SHR- and RD-STZ-SHR, respectively, p < 0.05), as well as the LF component of AP variability (1.62 ± 0.9, 2.12 ± 0.9, 7.38 ± 6.5 in RD-SHR, STZ-SHR and RD-STZ-SHR, respectively, p < 0.05). GLUT2 renal content was higher in all groups *vs*. SHR.

**Conclusions:**

Renal denervation in diabetic-hypertensive rats improved previously reduced heart rate variability. The GLUT2 equally overexpressed by diabetes and renal denervation may represent a maximal derangement effect of each condition.

## Background

Autonomic neuropathy is a common complication of diabetes mellitus that can be effectively reproduced in animal models of diabetes: studies performed in our laboratory in streptozotocin (STZ)-induced diabetic rats showed that these animals present long-lasting changes in blood pressure, heart rate and autonomic cardiovascular reflexes [1-4]. Hypertension, a common morbid condition associated with diabetes, can also have an impact on cardiovascular reflexes [5]. The induction of diabetes in spontaneously-hypertensive rats (SHR) enhances abnormalities usually seen in both animal models individually, i.e. spontaneous hypertension and diabetes: using spectral analysis approaches, we demonstrated decreased arterial pressure variability in diabetic SHRs [6]. Moreover, the well-documented impairment of heart rate baroreflex control previously observed in SHR is further depressed in these rats [6].

Renal denervation caused by autonomic neuropathy has been involved in the enhancement of kidney vulnerability to the hemodynamic effects of high blood pressure in diabetic nephropathy [7,8]. This is caused by decreased renal vascular resistance induced by decreased sympathetic nerve activity, so that there would be no opposition to diabetes and hypertension induced mesangial cell stretch (9). Even though the STZ-diabetic rat develops characteristic glomerulosclerotic lesions similar to those found in human diabetic nephropathy [9], this animal model does not develop systemic arterial hypertension [3], thus, the association of hypertension when SHR are made diabetic can approach this animal model to human diabetic nephropathy [10,11].

It has been suggested that increased expression of renal cortical GLUT1 (mesangial cells) [12], and GLUT2 (S1 tubular cells) [13] is involved in the development and progression of diabetic nephropathy. The well-known diabetes-induced GLUT2 overexpression and the further rise that can be caused by hypertension [13] in addition to hyperglycemia may lead to a further elevation in interstitial renal glucose concentration, and more glucose is taken up by mesangial cells through GLUT1. However, although we had already shown the effect of denervation upon GLUT2 [14], this has not yet been studied in diabetic-hypertensive rats.

Recently it was suggested that renal sympathetic innervation plays a role in the pathogenesis of diabetic nephropathy implying early enhancement in renal sensitivity to intrarenal norepinephrine on diabetic renal injury [15]. Since STZ-diabetes requires several months to determine structural autonomic denervation of the gastrointestinal tract [16] and of the heart [17], and no data are available on renal denervation induced by diabetes, we propose surgical renal denervation in SHR injected with STZ to evaluate the effects of renal denervation on cardiovascular reflexes and markers of diabetic nephropathy. The coexistence of diabetes and hypertension allows us to study the full effects of the failure of autonomic innervation on renal function in an animal model of diabetic nephropathy, with all its characteristics as they occur in humans. This study aimed at examining the effects of renal denervation upon mechanisms involved in the development of nephropathy (blood pressure levels, cardiovascular autonomic changes and renal glucose transporter GLUT2) in the STZ-SHR animal.

## Methods

### Animals

The investigation followed the ethical rules established by the *Guide for the Care and Use of Laboratory Animals *[18] and by the Colégio Brasileiro de Experimentação Animal (COBEA). The study was approved by the Research Ethics Committee of Instituto de Cardiologia do RS, protocol # UP: 3111/02. Experiments were performed on 41 male SHR, weighing 240-270 g. Sample size was calculated based on previous data from our group [11]. All animals were bred and kept under standard laboratory animal house conditions at the Animal Production and Research Unit of the Center for Scientific and Technological Development of Fundação Estadual de Produção e Pesquisa em Saúde do Rio Grande do Sul, Brazil. They were fed with standard balanced rat animal chow (12% protein content), given water ad libitum, and kept in special plastic cages with 3-4 rats exposed to a 12-hour light and 12-hour dark cycle (6 a.m./6 p.m.). Before all surgical procedures, rats were premedicated with 2 mg kg^-1 ^butorphanol.

### Diabetes induction of diabetes

Animals were acclimatized for 1 week, then fasted overnight and rendered diabetic (STZ-SHR) by a single injection of STZ (Sigma Chemical Co, St Louis, MO, USA), 50 mg/kg in the tail vein. Streptozotocin was dissolved in citrate buffer (pH 4.5) and injected slowly. Non-diabetic rats (SHR) were injected only with citrate buffer. Diabetes was defined as a non-fasting glucose >300 mg/dL in tail vein blood (test strips, Advantage, Roche, Brasil), 48 h after STZ injection. Glycemia was measured again at the end of all experiments.

### Renal denervation

Thirty days after STZ injection, bilateral renal denervation or sham surgery was performed in the 4 experimental groups: renal-denervated diabetic (RD-STZ-SHR, n = 11), diabetic (STZ-SHR, n = 11), renal-denervated hypertensive (RD-SHR, n = 10) and hypertensive (SHR, n = 9), under ketamine (90 mg/kg) and xylasine (10 mg/kg) anesthesia, i.p.. Renal denervation was accomplished by using a surgical-pharmacological procedure adapted as previously described and validated in our laboratory [14]. Mechanical denervation was performed by carefully stripping all visible nerves, at 16 × magnification (M90 stereomicroscope, FD Vasconcelos), along the renal arteries and veins from the aorta to the hilum of the kidney. Chemical denervation was performed by quickly painting the renal artery with 20% phenol in absolute ethanol. Then, the artery was washed with isotonic saline. For sham denervation, the surgical procedure was the same, but the renal artery and vein were not isolated and the nerves were left intact.

Fifteen days after the surgical procedure (45 days after the STZ injection) 24 h urine was collected in metabolic cages for glucose, sodium and albumin measurements.

After all protocols, the final sample size was: RD-STZ-SHR, n = 5, STZ-SHR, n = 6, RD-SHR, n = 6 and SHR, n = 7.

### Acquisition of cardiovascular variables

Catheters filled with saline were implanted into the femoral artery(PE-10), under ketamine and xylasine anesthesia for direct measurement of arterial pressure (AP). One day after catheter placement, the arterial cannula was connected to a strain-gauge transducer (P23Db, Gould-Statham, Oxnard, CA) and blood pressure signals were recorded during a 20-min period with a microcomputer equipped with an analog-to-digital converter board (CODAS, 2-kHz sampling frequency, Dataq instruments, Inc., Akron, OH). Rats were conscious and moved freely during the experiments. Recorded data were analyzed on a beat-to-beat basis.

### Assessment of autonomic control - Spectral analysis

Time series of pulse intervals (PI, tachograms) and systolic AP (SAP, systograms) were obtained from blood pressure records. Stationary fragments with about 300 beats, coincident in tachogram and systogram, were selected and spectral analysis was performed using an autoregressive model. The spectral bands for rats (very low frequency, VLF: 0.0-0.2 Hz, low frequency, LF: 0.2-0.75 Hz, high frequency, HF: 0.75-3.0 Hz) were defined according to previous references [19]. Tachogram and systogram spectra, for each stationary fragment, were evaluated quantitatively and values of HR variability (HRV) and SAP variability (SAPV) were obtained. Among the parameters obtained by spectral analysis, those distinguished for their physiological significance are the relationship between the powers of LF and HF components of HRV - the LF/HF index - or sympathetic-vagal balance [20], and the absolute powers of the LF and VLF components of SAPV, related to vascular sympathetic modulation and to renin-angiotensin system modulation on SAP [21]. Moreover, a relationship expressed by the root of the ratio between the absolute powers of LF components of HRV and VPAS - the LF alpha index - is known to express spontaneous baroreflex sensitivity [22].

### Renal harvesting

After cardiovascular evaluation (45 days of diabetes duration, 15 days after renal denervation), the rat kidneys were removed for cortical GLUT2 protein content evaluation. The rats were anesthetized with sodium pentobarbital (25 mg/kg body weight, *iv*), their kidneys were perfused with Hanks' buffer to eliminate the intravascular blood content, and removed. Renal outer cortex was carefully dissected and 1.5 mm slices were weighed and frozen at -70°C for further analysis.

### Laboratory measurements

Urinary glucose was measured using the colorimetric enzymatic test (commercial kit, Merck, Darmstadt, Germany, Centrifichem System 400-Roche/Cobas Mira-Roche). Urinary sodium concentrations were determined using an indirect ion selective electrode (ISE) together with the Mega-Bayer auto-analyzer. Samples for urinary albumin were collected without preservatives and stored at -70°C after centrifugation. Albuminuria was measured by a quantitative direct competitive enzyme-linked immunoabsorbant assay (ELISA) (Nephrat, Exocell Inc, Philadelphia, PA, USA) using a highly specific anti-rat albumin antibody. The quantification range for albuminuria was 0.156-10 mg/dl. Samples were diluted 1:2. Results were expressed in mg/24 h.

### GLUT2

Renal cortex GLUT2 protein expression was analyzed by Western blot following standard protocols with commercially acquired antibody (Chemicon International Inc., Temecula, CA, USA) [23]. Tissue samples were homogenized in 10 w/v buffer (10 mM) Tris-HCl, 1 mM EDTA, and 250 mM sucrose, pH 7.4, containing 5 mg/mL aprotinin, and centrifuged at 3000 *g *for 15 min. The supernatant was centrifuged at 12,000 *g *for 20 min, and the pellet was re-suspended as a plasma membrane enriched fraction (PM) [23]. Briefly, equal amounts of membrane protein (100 μg from medulla and 150 μg from cortex samples) were subjected to SDS-PAGE (10%) and transferred to nitrocellulose membrane by electrophoresis. After blocking with non-fat-milk, the sheets were incubated with the specific antiserum, followed by washing and incubation with (^125^I)-protein A (Amersham Biosciences UK Limited, Buckinghamshire, UK). After a final wash, the nitrocellulose sheets were dried at room temperature, and exposed to X-ray film for 5 days at -70°C. The blots were quantified by densitometric analysis, using the ImageQuant TLsoftware (Amersham Biosciences, Buckinghamshire, UK Pharmacia Biotech, Sweden). The results were normalized considering the mean of the values of control animals (SHR) in each membrane as 100, and reported as arbitrary units (AU). Loading control was performed by densitometry of Coomassie-stained gel [24].

All data obtained are reported in compliance with the ARRIVE guidelines on animal research [25].

### Statistical Analysis

Data are reported as means ± SEM. Statistical significance was analyzed by two-way analysis of variance (ANOVA), post-hoc Student-Newmann-Keuls. Urinary albumin data were log-transformed before analysis. Significance was defined at the 0.05 level. The Statistical Package for Social Sciences (version 15.0, SPSS, Chicago, Illinois) was used for data analysis.

## Results

General characteristics of the animals studied at baseline and 45 days after the STZ injection are shown in Table 1. Body weights were similar in all experimental groups at baseline (~266 ± 21 g, p = 0.729). Forty-five days after diabetes induction, STZ-SHR did not gain weight as the non-diabetic rats did. Therefore, the SHR and RD-SHR were heavier than STZ-SHR and RD-STZ-SHR at the end of the experiments. Renal denervation did not affect weight gain in any of the groups studied (p = 0.249).

**Table 1:** General characteristics of animals studied

	SHR (n = 9)	RD-SHR (n = 7)	STZ-SHR (n = 9)	RD-STZ-SHR (n = 5)	P (Denervation)	P (Diabetes)	P (Interaction)
Initial weight (g)	258 ± 14	267 ± 29	269 ± 13	272 ± 28	0.452	0.278	0.729
Weight 45 days after STZ injection (g)	310 ± 28	322 ± 35	275 ± 53*	223 ± 71#	0.249	<0.001	0.068
Initial plasma glucose (mg/dl)	84.4 ± 7	91.2 ± 5	414.1 ± 135*	357.4 ± 124#	0.463	<0.001	0.352
Plasma glucose 45 days after STZ injection (mg/dl)	92.0 ± 14	91.1 ± 9	417.5 ± 114*	388.0 ± 48#	0.548	<0.001	0.571
Diuresis (mL/24 h)	11.2 ± 4	20.7 ± 7	96.9 ± 42*	103.3 ± 38#	0.398	<0.001	0.872
24-h urinary glucose (mg/24 h)	0.37 ± 0.4	0.23 ± 0.4	758.0 ± 544*	1019.0 ± 379#	0.236	<0.001	0.235
24-h urinary sodium (mEq/24 h)	1.03 ± 0.3	1.29 ± 0.8	4.38 ± 2.1*	4.50 ± 1.8#	0.680	<0.001	0.881
24-h urinary albumin (mg)	152 [111-341]	711* [273-1304]	2456* [1491-3982]	1538# [667-2988]	0.472	<0.001	<0.001

Data are shown as mean ± SEM. SHR: spontaneously hypertensive rats; RD-SHR: renal-denervated spontaneously hypertensive rats; STZ-SHR: diabetic spontaneously hypertensive rats; RD-STZ-SHR: diabetic renal-denervated spontaneously hypertensive rats; n = indicates the number of animals. *P < 0.001 *vs. *H; #P < 0.01 *vs. *HRD; (Two-way ANOVA; post hoc: Student Newman Keuls). For the variables diuresis, urinary glucose, and urinary sodium the n is 9, 10, 11 and 11, respectively.

Glycemia was elevated ~4 fold, two and 45 days after STZ injection in STZ-SHR and RD-STZ-SHR when compared to SHR and RD-SHR groups (p < 0.001). Also, 24-h diuresis as well as 24-h urinary excretion of glucose, and sodium were higher in diabetic animals versus SHR and RD-SHR (p < 0.001) 45 days after STZ injection, confirming the severity of the diabetic state obtained. Renal denervation did not affect urinary glucose (p = 0.236), natriuresis (p = 0.680) and diuresis (0.398). Urinary albumin was higher in the diabetic groups (p < 0.001) *vs *SHR and RD-SHR and also higher in the HRD *vs *SHR group (p = 0.013). However, renal denervation did not cause additional albuminuria in RD-STZ-SHR as compared to STZ-SHR (Table 1).

Table 2 shows the cardiovascular parameters. Mean AP was ~20% lower in STZ SHR *vs. *SHR, with no difference among the other groups. Systolic and diastolic AP were lower in group STZ-SHR *vs*. SHR, P < 0.05. Diastolic AP was ~18% lower in group RD-STZ-SHR *vs. *RD-SHR (P < 0.05). Heart rate was ~ 12% higher in RD-SHR *vs*. SHR and ~18% lower in STZ-SHR *vs*. SHR. The RD-STZ-SHR group presented a lower heart rate as compared to the RD-SHR group and higher than the STZ-SHR group (p < 0.05).

**Table 2:** Cardiovascular parameters: Heart rate variability (HRV) and systolic arterial pressure variability (SAPV) analysis

	SHR (n = 7)	RD-SHR (n = 6)	STZ-SHR (n = 6)	RD-STZ-SHR (n = 5)
Heart rate (bpm)	384.82 ± 37.26	431.32 ± 36.60*	316.21 ± 5.17*	363.81 ± 12.25§#
Systolic AP (mmHg)	178.43 ± 16.07	171.03 ± 5.22	145.39 ± 8.49*	157.20 ± 26.41
Diastolic AP (mmHg)	141.17 ± 19.10	150.27 ± 13.52	116.26 ± 29.34*	118.88 ± 10.75#
Mean AP (mmHg)	160.13 ± 16.08	165.25 ± 8.89	129.22 ± 5.66*	139.90 ± 9.15#

HRV				

HRV (ms^2^)	69.84 ± 37.91	55.75 ± 25.21	73.40 ± 53.30	148.39 ± 93.58§#
LF peak (Hz)	0.64 ± 0.06	0.69 ± 0.08	0.55 ± 0.10	0.54 ± 0.14#
LF absolute (ms^2^)	5.17 ± 5.24	1.62 ± 0.98	2.12 ± 0.97	7.38 ± 6.52§#
LF nu	26.84 ± 19.05	26.07 ± 13.63	6.98 ± 3.86*	16.58 ± 7.99
HF (Hz)	2.42 ± 0.58	2.12 ± 0.82	2.00 ± 0.56	2.31 ± 0.65
HF absolute (ms^2^)	14.56 ± 12.83	4.14 ± 3.09	32.11 ± 15.88*	37.22 ± 20.54#
HF nu	73.16 ± 19.05	72.90 ± 12.59	93.02 ± 3.86*	83.42 ± 7.99
LF/HF Índex	0.47 ± 0.47	0.49 ± 0.32	0.08 ± 0.05*	0.21 ± 0.11

SAPV				

SAPV (mmHg^2^)	68.33 ± 36.16	61.89 ± 24.84	15.61 ± 10.26*	29.86 ± 20.69
VLF (mmHg^2^)	77.13 ± 54.27	38.00 ± 27.50	12.91 ± 8.11*	46.20 ± 47.03
LF (Hz)	0.44 ± 0.06	0.53 ± 0.06	0.36 ± 0.07*	0.40 ± 0.08
LF absolute (mmHg^2^)	18.49 ± 18.17	3.69 ± 3.10*	1.38 ± 1.38*	56.61 ± 26.75#
HF peak (Hz)	1.72 ± 0.68	2.12 ± 0.56	1.19 ± 0.22	1.52 ± 0.48
HF absolute (mmHg^2^)	4.67 ± 5.09	53.94 ± 17.65*	5.66 ± 5.45	6.27 ± 10.70#
α_LF _Índex	0.54 ± 0.16	0.86 ± 0.56	1.44 ± 0.38*	1.73 ± 1.38

Data are shown as mean ± SEM. SHR: spontaneously hypertensive rats; RD-SHR: renal denervated spontaneously hypertensive rats; STZ-SHR: diabetic spontaneously hypertensive rats; RD-STZ-SHR: diabetic renal-denervated spontaneously hypertensive rats; AP: arterial pressure; n = indicates the number of animals. VLF: very low frequency; LF: low frequency; HF: high frequency. *P < 0.05 *vs. *H, § P < 0.05 *vs. *D e #P < 0.05 *vs. *HRD. (Two-way ANOVA; post hoc: Student Newman Keuls).

Heart rate variability and SAPV are shown in table 2. Heart rate variability was similar between groups, but was higher in the RD-STZ-SHR *vs*. RD-SHR and STZ-SHR groups. The LF component of HRV, in absolute units (ms^2^), was increased by renal denervation in diabetic animals (p < 0.05, RD-STZ-SHR *vs*. RD-SHR and STZ-SHR group), accompanying the increase of total HRV. In normalized units (nu), the LF component was reduced by diabetes (STZ-SHR *vs*. SHR group, p < 0.05) which was offset in the diabetic group after renal denervation, since there was no difference among groups. The HF component of HRV in absolute units (ms^2^) was increased by diabetes (SHR *vs*. STZ-SHR group, p < 0.05, RD-SHR *vs*. RD-STZ-SHR, p < 0.05), with no reduction in hypertensive rats after renal denervation (SHR *vs*. RD-SHR group). In normalized units (nu), HF component was higher in diabetic rats (SHR *vs*. STZ-SHR group, p < 0.05), with no differences among the other groups. Sympathetic-vagal balance was decreased by diabetes (SHR *vs*. STZ-SHR group, p < 0.05), only in the non-denervated group. SAPV was reduced 4-fold by diabetes (SHR vs. STZ-SHR, p < 0.05), but did not differ among the other groups. The LF component of SAPV was shown to be diminished by diabetes and renal denervation (STZ-SHR and RD-SHR *vs*. SHR, p < 0.05) and increased when diabetes was associated with renal denervation (RD-STZ-SHR *vs*. RD-SHR, p < 0.05). The HF component of SAPV was enhanced by diabetes (SHR *vs*. STZ-SHR, p < 0.05, RD-SHR *vs. *RD-STZ-SHR, p < 0.05), with no difference observed between the SHR and RD-SHR groups. The alpha index, which estimates the spontaneous cardiac baroreflex sensitivity, was higher in STZ-SHR *vs*. SHR (P < 0.05).

Renal cortical GLUT2 was higher in RD-SHR, STZ-SHR and RD-STZ-SHR *vs*. SHR (99.9 ± 14, 185.6 ± 13, 182.7 ± 21, 201.4 ± 25 UA, p = 0.004). Thus, both renal denervation and diabetes caused GLUT2 overexpression, and their association did not induce additional changes.

## Discussion

The association of experimental hypertension, diabetes and surgical renal denervation can compose the picture we expect to see in long-term diabetes, as neuropathy and nephropathy are associated in a milieu where hyperglycemia and hypertension usually coexist. Here we showed the result of the association of the three conditions, concerning renal and autonomic variables in an animal model very close to long-standing human diabetes. The most important findings were: 1. surgical renal denervation did not change the baseline conditions of diabetes and hypertension significantly since the animals submitted to this procedure maintained high AP and glycemia; 2. heart rate and systolic AP variability were negatively affected by diabetes associated with hypertension, but renal denervation could compensate partially for these derangements; 3. diabetes and renal denervation could similarly cause overexpression of renal cortical GLUT2, with no additional effect of one condition upon the other; 4. albuminuria raised by the effect of diabetes and by the effects of renal denervation separately; high albuminuria already induced by diabetes/hypertension was not additionally affected by denervation in these animals.

### The animal model(diabetic-hypertensive)

It should first be mentioned that both the diabetic state induced by STZ (hyperglycemia, low body weight) and the hypertensive state (high AP levels) were maintained in renal-denervated rats, as we previously showed [13,14].

Although different from previous data from our group [11], the association of diabetes in the SHR slightly reduced mean AP, a finding that was previously reported by others [26], and could be explained by the low AP levels usually determined by STZ-diabetes [3]. However, AP values were still kept at hypertensive levels in the diabetic-SHR.

### Cardiovascular changes in renal denervated diabetic-hypertensive rats

Bradycardia was observed in diabetic rats, as previously described [1,3,4,6], but the interesting and new finding is that renal denervation increased the heart rate even in the diabetic-SHR. Almost all animal models of hypertension are characterized by hyperactivity of the sympathetic renal system [27,28]. Previous study by our group showed a heart rate reduction induced by bilateral renal denervation in hypertensive rats, but the hypertension was of short duration and was induced by aortic ligation [29]. In the present study, the tachycardia presented by RD-SHR was accompanied by a reduction in the sympathetic vascular modulation, which could be due to the lowered total vascular resistance previously described in renal-denervated SHR [30].

Analyzing heart rate variability using spectral analysis approaches, we observed that the LF component, in normalized units (nu), was reduced and the HF component, also in nu, of heart rate variability was elevated by diabetes, the former more than the latter. Thus the net effect was that the sympathovagal balance was reduced in these rats, which was also shown in non-hypertensive diabetic rats [31]. Again, renal denervation compensated this derangement, resulting in a sympathovagal balance almost at the levels of the controls (SHR). Concerning total heart rate variability, it was not reduced by diabetes, as we previously showed [6]; decreased heart rate variability was reported only after long periods of diabetes in normotensive rats [32-34]. Interestingly, again renal denervation was able to improve this cardiovascular derangement: diabetic-SHR denervated rats had the highest total heart rate variability. This was observed considering the comparisons of RD-STZ-SHR *vs *STZ-SHR and also RD-STZ-SHR *vs *SHR, showing an interaction between the diabetes and denervation factors in hypertensive rats. Although denervation could not change HRV in the nondiabetic rats, in diabetics this intervention was able to increase HRV. Heart rate variability is classically reduced in hypertension, diabetes and heart failure, resulting in additional cardiovascular risk to these individuals [19]. It is known that AP reduction can improve autonomic dysfunction in hypertensive individuals [35], but this is not the case, despite improved HRV, since RD-STZ-SHR and STZ-SHR had similar AP levels. The present results showed improved autonomic control by denervation in diabetic rats, a finding that supports the importance of the renal sympathetic nervous system in cardiac sympathetic and parasympathetic control.

On the other hand, the LF component of arterial pressure variability decreased in the diabetic-SHR, as previously shown in the same animal model [6] and in normotensive rats [3,4]. As the low frequency oscillations of blood pressure correspond to the influence of the sympathetic fibers acting on the cardiovascular system [36-38], this reduced sympathetic influence on blood vessels could explain, at least in part, the non-significant slightly lower blood pressure and its variability observed in the diabetic-SHR. Renal denervation raised arterial pressure variability, although not to the hypertensive group levels. Indeed, the changes in the LF component of systolic AP variability could result from changes in sympathetic outflow to different vascular beds in response to total nerve denervation, which includes abolishing afferent activity [39]. However, opposite effects of denervation upon the sympathetic influence on blood vessels occurred in hypertensive and hypertensive-diabetic rats. In the hypertensive group, denervation determined lower vascular sympathetic modulation, while in the diabetic group, vascular sympathetic modulation was raised. Accordingly, the reduction of AP observed only in diabetics may have contributed to these differences. Moreover, the effects of cardiopulmonary afferents on peripheral sympathetic activity, especially in the kidney, may have contributed to this increase in sympathetic modulation since in diabetic animals this reflex control is blunted [40]. Similarly, spontaneous baroreflex, evaluated by the α_LF _index was improved in diabetic rats, renal-denervated or not, probably accompanying their blood pressure levels, which were slightly lower than in the other rats. Baroreflex sensitivity was previously studied in STZ-induced diabetic rats, showing contradictory results, probably because of different diabetes duration [31,41] and evaluation methods [42].

### Renal changes in renal denervated diabetic-hypertensive rats

Renal cortical GLUT2, a marker of diabetes-induced renal injury [10,11] was raised by diabetes and denervation independently. These effects were not more intense when both conditions were associated (in the RD-STZ-SHR group), confirming previous data showing the sympathetic modulation of the expression of this glucose transporter [13]. The lack of additional regulation by the association of denervation/diabetes could be due to the fact that GLUT2 expression is already increased in SHR, as compared to Wistar rats [10], and diabetes and denervation induced further increase in GLUT2 expression, which might achieve such high maximal transcriptional and translational activity that the association could not increase it **any **more. GLUT2 changes were not accompanied by changes in urinary glucose, natriuresis and diuresis, as previously shown by us in normotensive rats [13,43,44], where we raised the possibility of a causal relationship between these variables. Again, this difference may be related to SHR, instead of Wistar rats, and further investigation of SGLT2 expression and cellular location of GLUT2 could clarify the relationship between GLUT2 and glycosuria and natriuresis. In addition, the absence of expected denervation diuresis and natriuresis in SHR [27] may be related to the increase in renal responsiveness to norepinephrine and angiotensin II under this condition in which the magnitude of renal nerve effects may be decreased [45].

An association between diabetic neuropathy and nephropathy by a possible effect of enhancing kidney vulnerability to the hemodynamic effects of blood pressure was suggested by a possible effect of denervation in determining an enhancement of kidney vulnerability to the hemodynamic effects of blood pressure [7]. However, renal denervation did not change albuminuria in diabetic rats, only in hypertensive nondiabetic rats. Thus, acceleration of diabetic nephropathy cannot be ascribed to it, as previously shown in normotensive rats [14], possibly because the amount of time rats were maintained denervated (only 14 days) could be insufficient to induce significant glomerular structural changes. Moreover, perhaps maximal injury was caused by hyperglycemia in diabetic rats, thus renal denervation could not exacerbate the glomerular damage which actually occurred in hypertensive rats not subjected to hyperglycemia, only to the hemodynamic effects of kidney vulnerability when there are high systemic levels of arterial pressure.

Interestingly, both GLUT2 and albuminuria followed the same direction in the animal model studied: they were raised by the diabetic and hypertensive states, and renal denervation did not have any additional effect upon both variables. Considering the following possible sequence of events previously suggested [13], high sympathetic tonus associated with hyperglycemia leads to overexpression of GLUT2, increased glucose reabsorption, higher interstitial concentrations of glucose, more glucose available to mesangial cells, GLUT1 overexpression [12], higher glucose uptake by mesangial cells and, finally, acceleration of the well-known intracellular steps involved in the pathogenesis of diabetic nephropathy.

Some limitations in the current study should be pointed out. Differences in sample size for different variables presented are related to the high mortality rates presented by these animals which was previously shown by others [46] and ascribed to the toxicity of STZ in addition to the hard surgical intervention (renal denervation) performed in the present study. It is possible that the healthiest rats survived, influencing the final results. Another possible limitation is that renal denervation could determine different results according to the animal model used, age and timing of the evaluation performed [6,29,31,41,42]. Indeed, some differences presented in this study as compared to others could be accounted for these differences.

## Conclusions

We conclude that the GLUT2 equally overexpressed by diabetes and renal denervation may represent a maximal derangement effect of each condition. The differences to previous studies could possibly be ascribed to the unique condition presented here, of diabetes associated with sympathetic hyperactivity and renal denervation. Renal denervation in this particular condition, which we think is the closest we have to the human condition, is characterized by the amelioration of previously reduced heart rate and heart rate variability, thus counterbalancing the previous high risk condition. As recently shown in a proof-of-principle study in patients with hypertension resistant to conventional therapy, where the renal denervation procedure proves to be safe and produces sustained lowering of arterial pressure [47], there could be other long-term benefits from the procedure, but they cannot yet be confirmed with the present results.

## Competing interests

The authors declare that they have no competing interests.

## Authors' contributions

LDD carried out the experimental studies, participated in the analysis and interpretation of data, statistical analysis, and manuscript writing. KRC carried out the signal processing, statistical analysis, interpretation of data and manuscript writing. NML and MSS carried out the experimental, metabolic and molecular analysis. FA carried out the experimental protocol and data records. MO carried out the metabolic and molecular analysis and interpretation of data and manuscript writing. UFM carried out the analysis and interpretation of data and manuscript writing. MCI participated in the interpretation of data and critical revision of manuscript. BDS participated in the conception and design of the study, carried out the analysis and interpretation of data and drafted the manuscript, revising it critically for important intellectual content. All authors read and approved the final manuscript.
