# Correction: Renal denervation in an animal model of diabetes and hypertension: Impact on the autonomic nervous system and nephropathy

**DOI:** 10.1186/1475-2840-10-49

**Published:** 2011-06-07

**Authors:** Lucinara D Dias, Karina R Casali, Natalia M Leguisamo, Felipe Azambuja, Martina S Souza, Maristela Okamoto, Ubiratan F Machado, Maria Cláudia Irigoyen, Beatriz D Schaan

**Affiliations:** 1 Instituto de Cardiologia do Rio Grande do Sul/Fundação Universitária de Cardiologia (IC/FUC), Porto Alegre, Brazil; 2 Universidade Federal do Rio Grande do Sul, Endocrine Division HCPA, Porto Alegre, Brazil; 3 Institute of Biomedical Scientes, University of São Paulo, São Paulo, Brazil; 4 Instituto do Coração (INCOR), São Paulo, Brazil

## Correction

After publication of this work [1], we noted an error in the results section of the abstract. The results for heart rate and the LF component of AP variability for the SHR sample were omitted from the list. The corrected results section appears below.

## Results

Higher glycemia (p < 0.05) and lower mean AP were observed in diabetics vs. nondiabetics (p < 0.05). Heart rate was higher in renal-denervated hypertensive and lower in diabetic-hypertensive rats (384.8 +/- 37, 431.3 +/- 36, 316.2 +/- 5, 363.8 +/- 12 bpm in SHR, RD-SHR, STZ-SHR and RD-STZ-SHR, respectively). Heart rate variability was higher in renal-denervated diabetic-hypertensive rats (69.84 ± 37.91, 55.75 ± 25.21, 73.40 ± 53.30, 148.4 ± 93 in SHR, RD-SHR, STZ-SHR- and RD-STZ-SHR, respectively, p < 0.05), as well as the LF component of AP variability (5.17 ± 5.24, 1.62 ± 0.9, 2.12 ± 0.9, 7.38 ± 6.5 in SHR, RD-SHR, STZ-SHR and RD-STZ-SHR, respectively, p < 0.05). GLUT2 renal content was higher in all groups vs. SHR.
